# Feasibility of iodine concentration and extracellular volume fraction measurement derived from the equilibrium phase dual-energy CT for differentiating thymic epithelial tumors

**DOI:** 10.1007/s11604-022-01331-9

**Published:** 2022-08-27

**Authors:** Koji Takumi, Hiroaki Nagano, Tsuyoshi Myogasako, Tsubasa Nakano, Yoshihiko Fukukura, Kazuhiro Ueda, Kazuhiro Tabata, Akihide Tanimoto, Takashi Yoshiura

**Affiliations:** 1 Department of Radiology, Kagoshima University Graduate School of Medical and Dental Sciences, 8-35-1 Sakuragaoka, Kagoshima, 890-8544 Japan; 2 Department of General Thoracic Surgery, Kagoshima University Graduate School of Medical and Dental Sciences, 8-35-1 Sakuragaoka, Kagoshima, 890-8544 Japan; 3 Department of Human Pathology, Kagoshima University Graduate School of Medical and Dental Sciences, 8-35-1 Sakuragaoka, Kagoshima, 890-8544 Japan

**Keywords:** Thymic epithelial tumor, Dual-energy computed tomography, Extracellular space, Iodine quantification

## Abstract

**Purpose:**

To assess the diagnostic feasibility of iodine concentration (IC) and extracellular volume (ECV) fraction measurement using the equilibrium phase dual-energy CT (DECT) for the evaluation of thymic epithelial tumors (TETs).

**Materials and methods:**

This study included 33 TETs (11 low-risk thymomas, 11 high-risk thymomas, and 11 thymic carcinomas) that were assessed by pretreatment DECT. IC was measured during the equilibrium phases and ECV fraction was calculated using IC of the thymic lesion and the aorta. IC and ECV fraction were compared among TET subtypes using the Kruskal–Wallis *H* test and Mann–Whitney *U* test. Receiver-operating characteristic (ROC) curve analysis was performed to evaluate the ability of IC and ECV fraction to diagnose thymic carcinoma.

**Results:**

IC during the equilibrium phase and ECV fraction differed among the three TET groups (both *p* < 0.001). IC during the equilibrium phase and ECV fraction was significantly higher in thymic carcinomas than in thymomas (1.9 mg/mL vs. 1.2 mg/mL, *p* < 0.001; 38.2% vs. 25.9%, *p* < 0.001; respectively). The optimal cutoff values of IC during the equilibrium phase and of ECV fraction to diagnose thymic carcinoma were 1.5 mg/mL (AUC, 0.955; sensitivity, 100%; specificity, 90.9%) and 26.8% (AUC, 0.888; sensitivity, 100%; specificity, 72.7%), respectively.

**Conclusion:**

IC and ECV fraction measurement using DECT are helpful in diagnosing TETs. High IC during the equilibrium phase and high ECV fraction are suggestive of thymic carcinoma.

## Introduction

Thymic epithelial tumors (TETs) are the most common primary neoplasm of the anterior mediastinum and have heterogeneous oncologic behaviors and variable histologic features [1]. Clinically, TETs can be divided into three groups based on the World Health Organization (WHO) classification: low-risk thymomas (type A, AB, and B1), high-risk thymomas (type B2 and B3), and thymic carcinomas. TET group is an independent prognostic factor of survival in patients with TETs [2]. Low-risk thymomas are usually treated with surgery, and have a lower tumor recurrence rate and higher survival rate compared with high-risk thymomas and thymic carcinomas, which generally undergo multimodality treatment [3]. Approximately 30% of TETs are diagnosed as advanced staged (Masaoka-Koga III–IVa) and may not undergo complete resection due to the local extent [4]. Debulking surgery for advanced unresectable thymoma can improve overall survival [5]. However, a previous study did not find any significant differences in survival between patients with thymic carcinomas who underwent debulking surgery and those who underwent no surgery [6]. Therefore, an accurate pretreatment diagnosis of the TET subtype is valuable for physicians.

Core needle biopsy is a well-established and useful procedure for diagnosing mediastinal tumors. However, international thymic malignancy interest group (ITMIG) recommends caution in classifying WHO subtype on a fine needle aspiration (FNA) or biopsy specimen, because TETs can be histologically quite heterogeneous, with 30–50% showing some histology found in a single tumor [1, 7]. According to the National Comprehensive Cancer Network (NCCN) guidelines for TETs, contrast-enhanced chest CT remains the first choice for pretreatment imaging evaluation of TETs [8]. Many studies have reported the CT imaging findings of TETs [9–15]. The presence of irregular contour, necrotic or cystic component, heterogeneous enhancement, lymphadenopathy, and great vessel invasion are suggestive of thymic carcinoma [9]. However, there is considerable overlap in the conventional CT imaging features among the histological subtypes [10, 11].

WHO classification of TETs is determined pathologically based on the morphologic manifestations of epithelial cells and the ratio of lymphocytes to epithelial cells [1], which can impact tissue characteristics such as cellularity, vascularity, and the amount of extracellular matrix. The radiographic contrast enhancement characteristics are useful for assessing the tissue composition of lesions and have been reported to be useful for evaluating TETs [14, 16, 17]. Some recent studies have reported that iodine concentration (IC) map derived from dual-energy CT (DECT) provides information about angiogenesis, and its usefulness has also been shown for evaluating lesions in various organs [18–21]. Therefore, it is conceivable that IC measurement could be applied to differentiating TET subtypes. IC has been reported to be significantly different between subtypes of TET [20]. ICs for low-risk thymomas were significantly higher during the arterial and venous phases compared to those for high-risk thymomas and thymic carcinomas [21]. There has been no study of the diagnostic ability of IC during the equilibrium phase for evaluating TETs. A recent study has suggested that IC during the equilibrium phase of DECT can improve the diagnosis of myocardial fibrosis by calculating the extracellular volume (ECV) fraction, and that myocardial ECV fraction derived from the equilibrium phase showed a close correlation with the MR imaging findings [22]. Chang et al. reported the usefulness of ECV fraction derived from MR T1 mapping for predicting WHO classification of TETs [23]. The ECV fractions of lymphocyte-sparse TETs such as type A and B3 thymomas and thymic carcinomas were significantly higher than those of lymphocyte-abundant TETs such as type AB, B1, and B2 thymomas. We hypothesized that IC and ECV fraction derived by DECT would correlate with the WHO classification of TETs. Therefore, the purpose of this study was to assess the diagnostic feasibility of IC and ECV fraction measurement using the equilibrium phase DECT for the evaluation of TET subtype.

## Materials and methods

### Patients

Institutional ethics review board approval was obtained and informed consent was waived for this retrospective study. Patients who were treated for TET at our hospital and who underwent pretreatment DECT between January 2017 and December 2020 and who met the following inclusion criteria were enrolled in the study: (a) pathologically confirmed TET, (b) lesions larger than 10 mm in diameter, and (c) no history of biopsy or treatment for thymic lesions before the CT examination. Excluded from the study were patients with imaging of inadequate quality and those with an interval of more than one week between the CT exam and hematocrit level measurement for ECV fraction calculation.

### DECT examination

CT examination was performed using a 64 multi-detector row dual-layer DECT (IQon spectral CT; Philips Healthcare, Best, the Netherlands). All patients were scanned in the supine position with hands raised above the head. The imaging parameters were as follows: tube voltage, 120 kVp; effective tube current–time product, 160 mAs with auto-modulation; gantry rotation time, 0.4 s; pitch, 0.703; and detector row configuration, 64 × 0.625 mm. Equilibrium phase scans was obtained at delays of 280 s after aortic enhancement exceeded 150 Hounsfield units (HU) compared with baseline. Nonionic contrast agent (Omnipaque 300 mgI/mL; Daiichi Sankyo, Tokyo, Japan) was injected at a dose of 2.0 mL/kg body weight (fixed at 150 mL for patients weighing ≥ 75.0 kg) using a power injector at a fixed duration of 30 s. Conventional 120 kVp images and IC maps were reconstructed in the axial plane with slice thickness of 1 mm.

### DECT image analysis

Imaging analysis was performed using a thin-client workstation (Spectral Diagnostic Suite; Philips Healthcare). All images were independently evaluated by two radiologists (with 20 and 5 years of experience in chest radiology) who were blinded to the final pathological results. The following CT morphological features of the thymic lesions were evaluated: size (long and short diameters), shape (round/lobulated/irregular), boundary (well-defined/ill-defined), homogeneity (homogeneous/heterogeneous), necrotic or cystic change (presence/absence), calcification (presence/absence), lymphadenopathy (presence/absence), and pleural effusion (presence/absence). Necrotic or cystic change was defined as a hypoattenuating lesion < 30 HU during the equilibrium phase of enhanced CT. Lymphadenopathy was judged to be present in the case of short diameter > 10 mm or the presence of intranodal necrotic change. All morphological features were evaluated on conventional 120 kVp images. Adjustment of the window level and width was permitted during qualitative assessment. Any discrepancies between the radiologists’ qualitative evaluations were discussed and resolved by consensus. Each radiologist also manually placed a freehand region-of-interest (ROI) within the lesion on the slice showing the largest area of the lesion on each of the IC maps. A circular ROI of area 50 mm^2^ was also placed on the aorta in the equilibrium phase. Care was taken to avoid including artifact or necrotic/cystic change within the ROIs. ECV fraction (%) was calculated using the following formula:

$${\text{ECV}}\;{\text{fraction}}\;(\% ) = \left( {1 - {\text{hematocrit}}} \right) \times \left( {{\text{IC}}_{{{\text{Lesion}}}} /{\text{IC}}_{{{\text{Aorta}}}} } \right) \times 100,$$

where IC_Lesion_ and IC_Aorta_ are ICs (mg/mL) during the equilibrium phase for the thymic lesion and the aorta, respectively. The mean value of the two radiologists was obtained as the representative value of each tumor for all quantitative variables.

### Pathological diagnosis

The final diagnosis was determined by histological examination of surgical specimens. Resected tissues were fixed with 10% neutral phosphate-buffered formalin, routinely processed for paraffin embedding, sectioned for hematoxylin and eosin (HE) staining. Pathological diagnoses were made by two board-certified pathologists according to WHO classifications. All TET lesions were categorized according to the six histologic subtypes of the 2015 WHO histological classification, and then divided into the following three groups: low-risk thymoma (types A, AB, and B1), high-risk thymoma (types B2 and B3), and thymic carcinoma. TETs were additionally divided into the following two groups: low-risk thymic epithelial tumor (LRTET) including low-risk thymoma, and high-risk thymic epithelial tumor (HRTET) including high-risk thymoma and thymic carcinoma.

### Statistical analysis

Interobserver agreement for all qualitative analyses was evaluated using weighted kappa analysis (0.00–0.20, slight; 0.21–0.40, fair; 0.41–0.60, moderate; 0.61–0.80, substantial; 0.81–1.00, excellent). Interobserver agreement for quantitative measurements was evaluated using intraclass correlation coefficient (ICC) (0.00–0.20, poor correlation; 0.21–0.40, fair correlation; 0.41–0.60, moderate correlation; 0.61–0.80, good correlation; 0.81–1.00, excellent correlation). Shape, boundary, homogeneity, necrotic or cystic change, calcification, lymphadenopathy, and pleural effusion were evaluated using the chi-squared test and Fisher's exact test for comparison among the low-risk thymoma, high-risk thymoma, and thymic carcinoma groups followed by post hoc test; between LRTET and HRTET; and between all thymomas and thymic carcinomas. Size, long-to-short axis ratio, IC, and ECV fraction were compared using the Kruskal–Wallis *H* test or Mann–Whitney *U* test. Receiver-operating characteristic (ROC) curve analysis was performed to evaluate the ability of continuous values to diagnose HRTET or thymic carcinoma. Area under the ROC curves (AUC) of IC and ECV were compared using DeLong’s test. Sensitivity and specificity were calculated using a threshold criterion that would maximize the Youden index. All data for continuous variables are presented as mean ± standard deviation (SD). Values of *p* < 0.05 and *p* < 0.017 (Bonferroni correction) were considered indicative of significance in all analyses. Statistical analyses were performed using MedCalc version 19.6 (MedCalc Software, Mariakerke, Belgium) and SPSS version 25.0 (SPSS, Chicago, IL).

## Results

### Patients and thymic tumor classifications

**Fig. 1:**
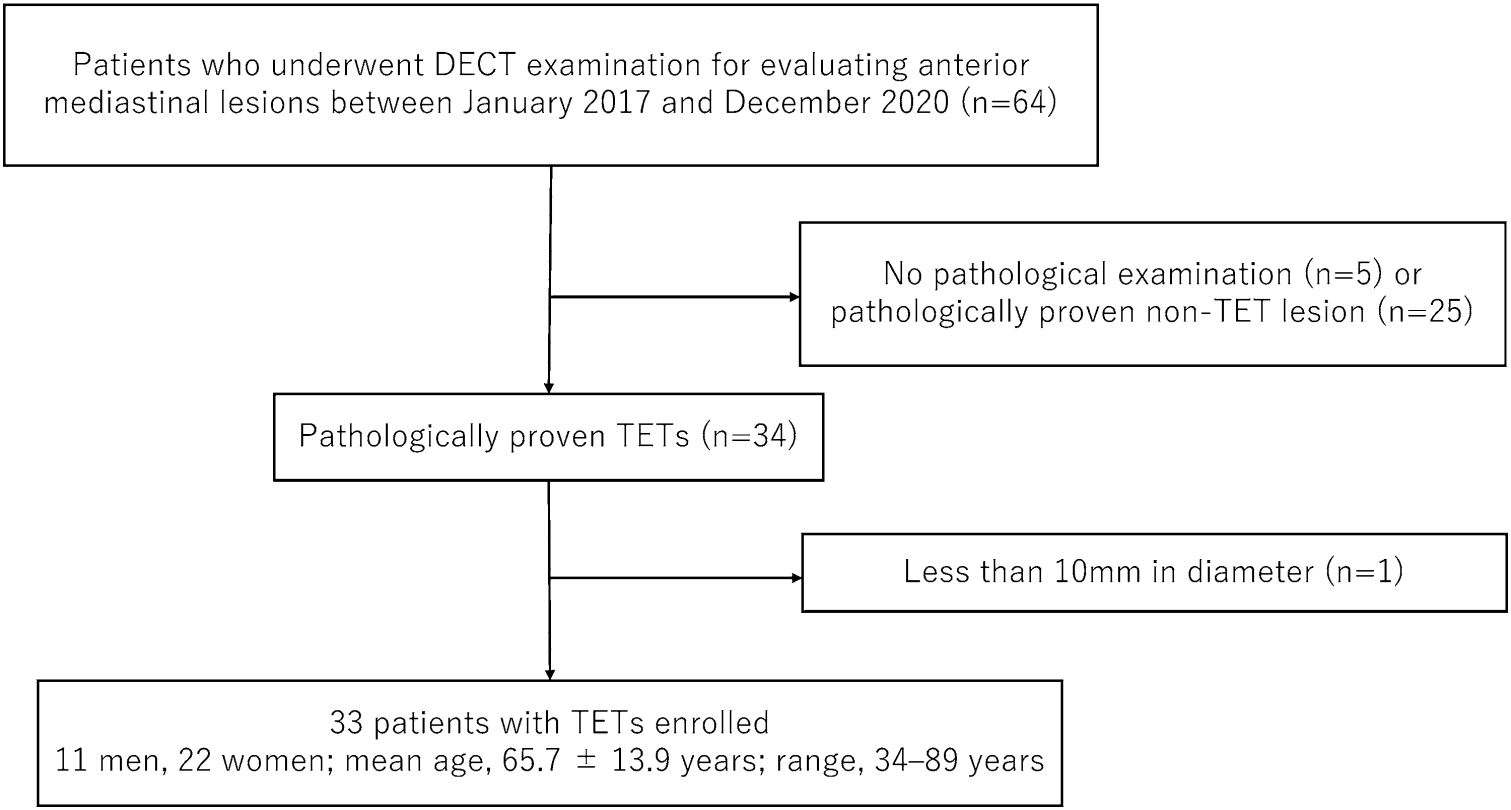
Flow diagram of the study population. *TET* thymic epithelial tumor, *DECT* dual-energy CT

Identified and included in the study were 33 consecutive patients (11 men, 22 women; mean age, 65.7 ± 13.9 years; range 34–89 years) (Fig. 1). Table 1 summarizes the clinical and pathological characteristics of the study population. The pathological diagnoses of TETs were type A (*n* = 0), type AB (*n* = 4), type B1 (*n* = 7), type B2 (*n* = 6), type B3 (*n* = 5), and thymic carcinoma (*n* = 11).

**Table 1:** Clinical and demographic characteristics of 33 patients with thymic epithelial tumors

Variables	N = 33
Gender (M:F)	11:22
Age (mean ± SD)	65.7 ± 13.9
Major clinical symptoms	
Pain or pressure in the chest	4
Shortness of breath	4
Fatigue	3
Facial swelling	2
Fever	2
Drooping eyelids	1
No symptom	17
Mosaoka-Koga stage	
I	3
II	21
III	1
IV	8
WHO classification	
A	0
AB	4
B1	7
B2	6
B3	5
Carcinoma	11

### Interobserver agreement

Interobserver agreement ranged from substantial to excellent for qualitative parameters (*κ* = 0.95 for shape, *κ* = 0.82 for boundary, *κ* = 0.76 for homogeneity, *κ* = 0.94 for necrotic or cystic change, *κ* = 0.68 for calcification, *κ* = 1.00 for lymphadenopathy, *κ* = 0.78 for pleural effusion) and were excellent for all quantitative measurements (ICC = 0.99 for long diameter, ICC = 0.99 for short diameter, ICC = 0.96 for IC of TET, ICC = 0.99 for IC of aorta, ICC = 0.90 for ECV fraction).

### Imaging parameters among the three TET groups

Table 2 lists the DECT features among the three groups (low-risk thymomas, high-risk thymomas, and thymic carcinomas). Shape, boundary, homogeneity, necrotic or cystic changes, lymphadenopathy, pleural effusion, IC, and ECV fraction showed significant difference among the three groups (all *p* < 0.05), whereas long and short diameters, and calcification showed no significant difference among the groups (all *p* > 0.05). Shape, boundary, necrotic or cystic changes, lymphadenopathy, IC, and ECV fraction showed significant differences between low-risk thymoma and carcinomas (all *p* < 0.017), and lymphadenopathy, IC, and ECV fraction showed significant differences between high-risk thymoma and carcinomas (all *p* < 0.017). However, there was no significant difference between low- and high-risk thymomas (all *p* > 0.017).

**Table 2:** Comparison of clinical and CT imaging features among subgroups of thymic epithelial tumors

	Subtypes of thymic epithelial tumors				*p*				
	Low-risk thymomas (*n* = 11)	High-risk thymomas (*n* = 11)	Thymic carcinomas (*n* = 11)	Among three groups	Low-risk vs. high-risk thymomas	Low-risk thymomas vs. carcinomas	High-risk thymomas vs. carcinomas	LRTETs vs. HRTETs	Thymomas vs. carcinomas
Age	60.9 ± 15.4	67.7 ± 12.2	68.4 ± 13.9	0.392	1.000	0.930	1.000	0.169	0.440
Gender (M:F)	3: 8	3: 8	5: 6	0.580	1.000	0.659	0.659	1.000	0.437
Histological types	AB: 4	B2: 6	Carcinoma: 11						
	B1: 7	B3: 5							
Long diameter (mm)	41.1 ± 17.3	54.5 ± 26.6	47.1 ± 13.0	0.299	0.885	0.885	1.000	0.193	0.929
Short diameter (mm)	21.9 ± 11.5	34.5 ± 20.4	28.7 ± 13.2	0.176	0.603	0.149	1.000	0.098	0.936
Shape				0.019*	0.118	0.005**	0.135	0.009*	0.027*
Round	7	3	0						
Lobulated	1	5	5						
Irregular	3	3	6						
Boundary				0.003*	0.183	0.002**	0.149	0.008*	0.004*
Well-defined	9	5	1						
Ill-defined	2	6	10						
Homogeneity				0.021*	1.000	0.024	0.063	0.136	0.009*
Homogeneous	7	5	1						
Heterogeneous	4	6	10						
Necrotic/cystic change				0.010*	0.387	0.008**	0.149	0.024*	0.009*
Presence	3	6	10						
Absence	8	5	1						
Calcification				0.281	0.311	1.000	0.635	0.378	1.000
Presence	1	4	2						
Absence	10	7	9						
Lymphadenopathy				0.001*	1.000	0.012**	0.012**	0.077	< 0.001*
Presence	0	0	5						
Absence	11	11	6						
Pleural effusion				0.037*	1.000	0.214	0.214	0.534	0.030*
Presence	0	0	3						
Absence	11	11	8						
Iodine concentration (mg/mL)	1.2 ± 0.2	1.2 ± 0.3	1.9 ± 0.3	< 0.001*	1.000	< 0.001**	0.002**	0.082	< 0.001*
ECV fraction (%)	24.5 ± 4.4	27.3 ± 9.7	38.2 ± 7.6	< 0.001*	1.000	0.002**	0.014**	0.021*	< 0.001*

*LRTETs* low-risk thymic epithelial tumors, *HRTETs* high-risk thymic epithelial tumors, *ECV* extracellular volume

*Significantly different (*p* < 0.050)

**Significantly different (*p* < 0.017)

*P*-values marked with bold indicate statistically significant differences

### Comparison between LRTETs and HRTETs

**Fig. 2:**
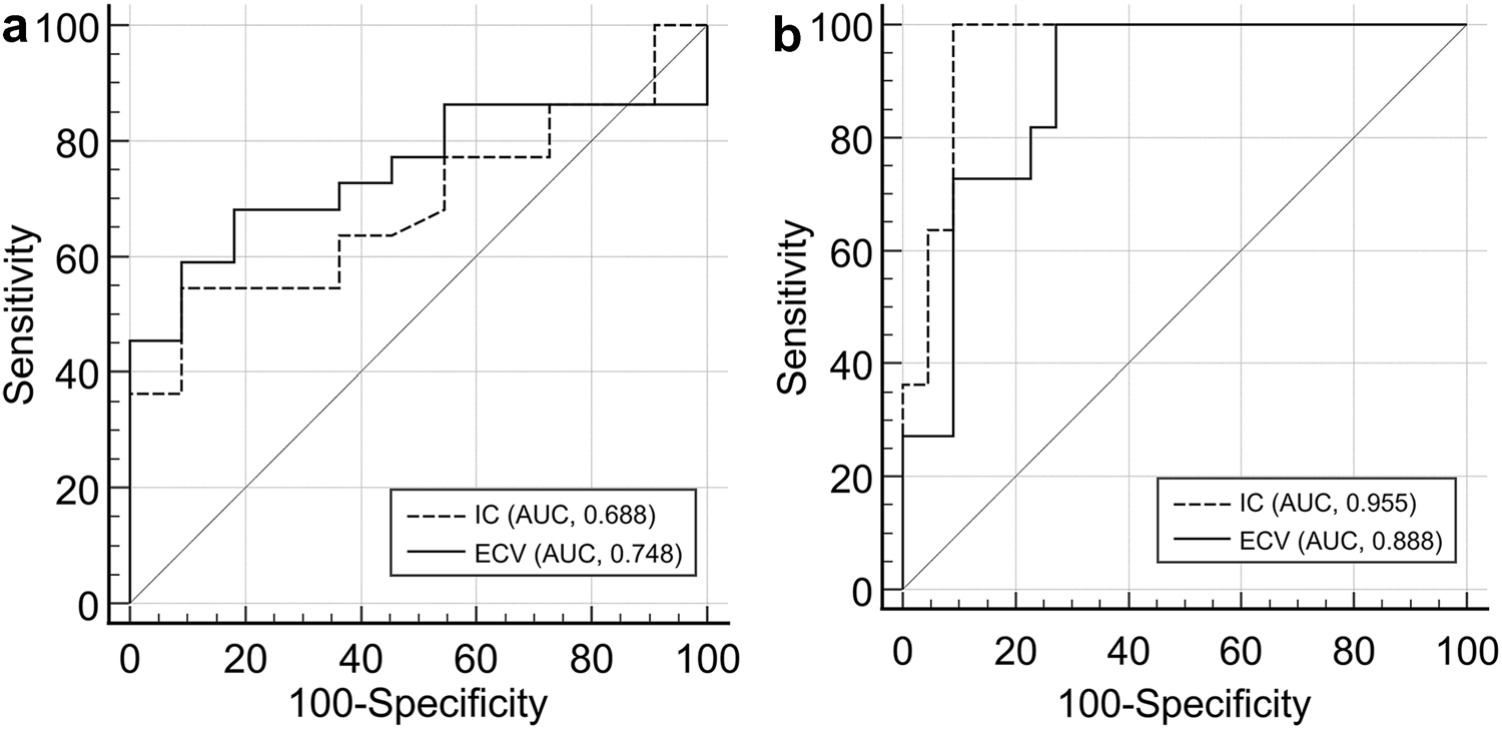
Receiver-operating characteristic (ROC) curves for diagnosis of high-risk thymic epithelial tumors (**a**) and thymic carcinomas (**b**) with iodine concentration (IC) during the equilibrium phase and extracellular volume (ECV) fraction. No significant difference is identified between the area under the ROC curve (AUC) of IC during the equilibrium phase and that of ECV fraction for diagnosing high-risk thymomas and thymic carcinomas (*p* = 0.336 and 0.149, respectively)

Lobulated or irregular shape, ill-defined boundary, and necrotic or cystic change were significantly more frequent in HRTETs than in LRTETs (all *p* < 0.05). ECV fraction was significantly higher in HRTETs than in LRTETs (32.8% vs. 24.5%, *p* = 0.021). The optimal cutoff value of IC and ECV fraction for differentiating HRTET from LRTET were 1.5 mg/mL (AUC, 0.688 with 95% CI of 0.504–0.837; sensitivity, 54.5% with 95% CI of 32.2 to 75.6; specificity, 90.9% with 95% CI of 58.7–99.8) and 26.8% (AUC, 0.748 with 95% confidence interval [CI] of 0.56–0.882; sensitivity, 68.2% with 95% CI of 45.1–86.1; specificity, 81.8% with 95% CI of 48.2–97.7), respectively (Fig. 2a). No significant difference is identified between AUC of IC and that of ECV fraction (*p* = 0.336).

### Comparison between thymomas and thymic carcinomas

Compared with all thymomas, lobulated or irregular shape, ill-defined boundary, intralesional heterogeneity, necrotic or cystic change, lymphadenopathy, and pleural effusion were significantly more frequent in thymic carcinomas (all *p* < 0.05). IC and ECV fraction were significantly larger in thymic carcinomas than in thymomas (1.9 mg/mL vs. 1.2 mg/mL, *p* < 0.001; 38.2% vs. 25.9%, *p* < 0.001; respectively). The optimal cutoff values of IC and ECV fraction to differentiate thymic carcinomas from all thymomas were 1.5 mg/mL (AUC, 0.955 with 95% CI of 0.819–0.997; sensitivity, 100% with 95% CI of 71.5–100.0; specificity, 90.9% with 95% CI of 70.8–98.9) and 26.8% (AUC, 0.888 with 95% CI of 0.730–0.971; sensitivity, 100% with 95% CI of 71.5–100.0; specificity, 72.7% with 95% CI of 49.8–89.3), respectively (Fig. 2b). No significant difference is identified between AUC of IC and that of ECV fraction (*p* = 0.149).

**Fig. 3:**
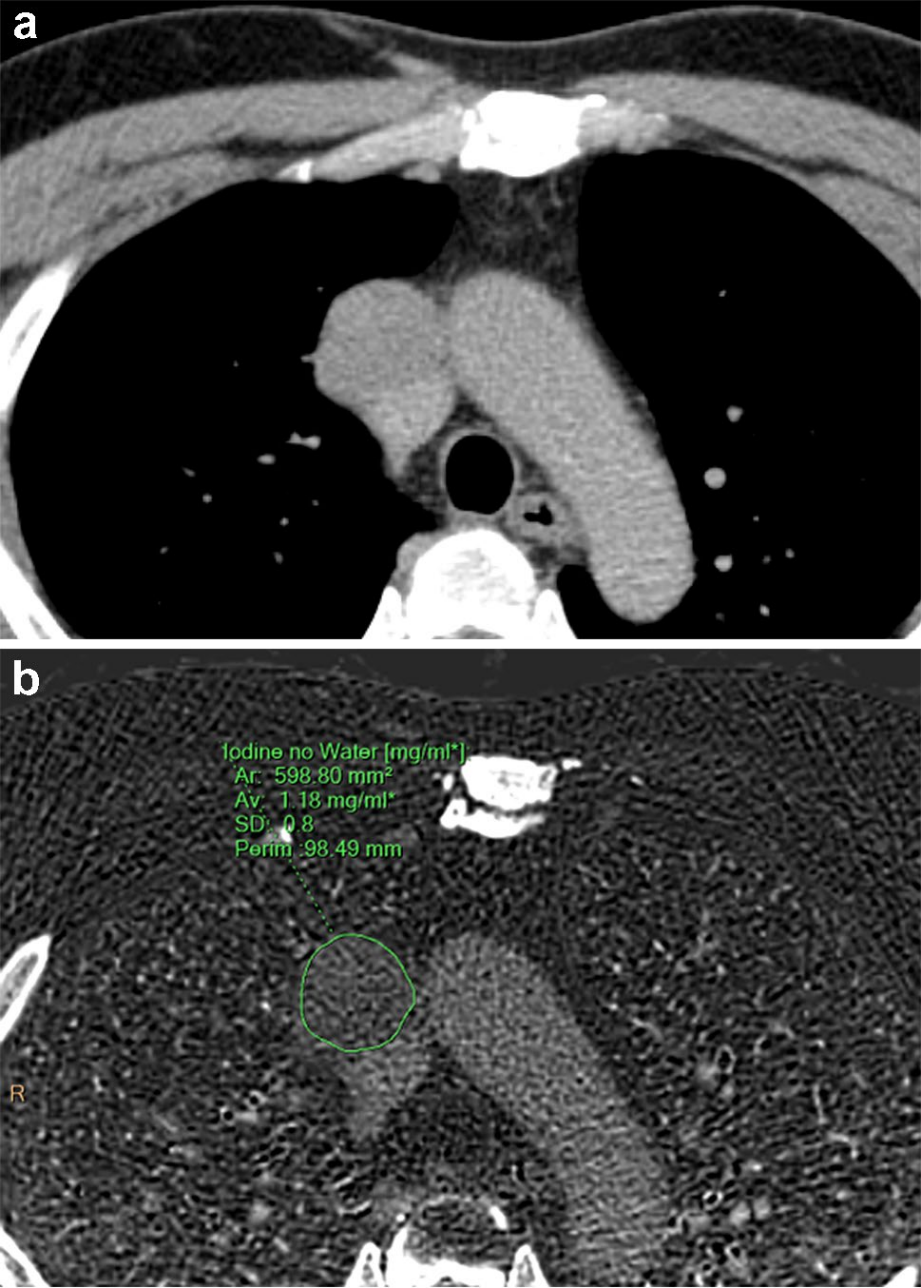
A 39-year-old male with low-risk thymoma (type AB). Contrast enhanced CT (**a**) shows a homogeneous, rounded, and well-defined tumor in the right anterior mediastinum. Iodine concentration during the equilibrium phase (**b**) and extracellular volume fraction are 1.18 mg/mL and 25.6%, respectively

**Fig. 4:**
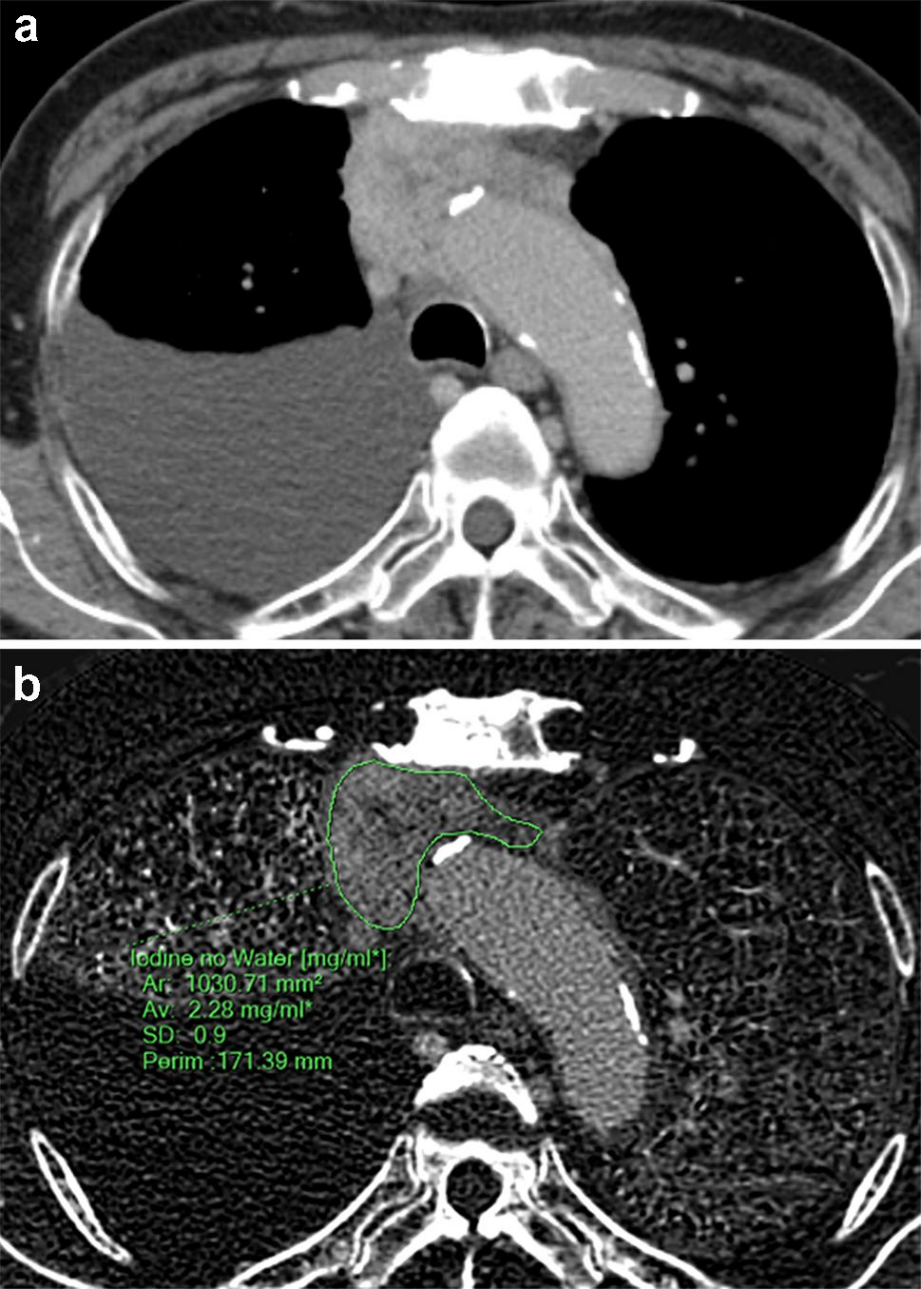
An 89-year-old male with thymic carcinoma. Contrast enhanced CT (**a**) shows a heterogeneously enhancing, irregularly shaped and ill-defined tumor in the anterior mediastinum, with pleural effusion. Iodine concentration during the equilibrium phase (**b**) and extracellular volume fraction are 2.28 mg/mL and 40.8%, respectively

Representative cases are shown in Figs. 3, 4.

## Discussion

The present study demonstrated the diagnostic potentials of IC and ECV fraction derived from DECT analysis for evaluating pathological subtype of TETs. ECV fraction was significantly higher in HRTETs than in LRTETs. IC during the equilibrium phase and ECV fraction were significantly higher in thymic carcinomas than in thymomas.

In agreement with those of prior studies, the present results showed that irregular margin, mediastinal fat invasion, intralesional heterogeneity, lymphadenopathy, and pleural effusion were more commonly seen in HRTETs compared with LRTETs [12–14]. Contrast enhancement features have also been reported as useful in evaluating TETs [14, 16, 17]. A previous CT study showed that maximal contrast enhancement was significantly higher in type A and type AB thymomas than in other types of TETs [14]. DECT findings of TETs have been reported in several recent studies [20, 21]. The slope of the spectral Hounsfield unit curve of virtual monochromatic images and IC were found to be significantly different among TET subtypes [20]. ICs of low-risk thymomas were significantly higher during the arterial and venous phases compared with those of high-risk thymomas and thymic carcinomas [21]. In the present study, IC during the equilibrium phase was significantly higher in thymic carcinomas than in all thymomas. The opposite tendency of IC within the lesion on the equilibrium phase compared to that on the arterial or venous phases can be explained by the difference in the dynamic contrast enhancement pattern of TET. Low-risk thymomas typically show washout pattern, whereas high-risk thymoma and thymic carcinoma more frequently show persistent or plateau patterns [16]. Therefore, the difference in scan timing may have affected the result of the IC value. This is the first report to examine the diagnostic ability of IC during the equilibrium phase for evaluating TETs. The equilibrium phase corresponds to the approximate time span required for the plasma–contrast concentration to become similar or equal to the interstitial concentration. The equilibrium phase images may enable assessment of stromal condition within a lesion, and could be particularly useful for evaluating lesions in which the stromal features vary by histological subtype, such as TETs.

ECV fraction measurement has been reported as useful for assessing and quantifying tissue composition, and recent studies have provided good evidence for an association between elevated ECV fraction during the equilibrium phase and diffuse fibrosis in the liver and heart [18, 24, 25]. ECV fraction measurement may enable dichotomization of TETs into cellular and extracellular components, providing valuable information for pathologic correlation. The pathological subtypes of TETs according to the WHO classification are characterized by the content of neoplastic epithelial cells and nonneoplastic immature T-cells. Low-risk thymomas, especially types AB and B1, contain an abundance of immature T-cells within the lesion that could result in high cellularity with a decrease in the extracellular space. In contrast, high-risk thymomas and thymic carcinomas show a paucity or lack of immature T-cells, which could result in high extracellular space compared with low-risk thymomas. In the present study, the ECV fraction was significantly higher in HRTETs and thymic carcinomas than in LRTETs and thymomas. The present results are in line with those of a recent MR imaging study that revealed a correlation between ECV fraction calculated by T1 mapping and the WHO histologic classification of TETs [23]. However, mediastinal MR images are commonly degraded by severe imaging artifacts such as pulsation, motion artifacts from the heart, and magnetic susceptibility distortion artifact, which limits the reproducibility of quantitative MR parameters, especially for small-sized lesions. Moreover, ECV fraction measurement using MR T1 mapping requires pre- and post-contrast images and misregistration between these images may reduce reproducibility and diagnostic accuracy. DECT has some advantages compared with MR imaging, as it is capable of assessing mediastinal lesions with shorter examination time, lower cost, higher spatial resolution, and fewer artifacts, which may improve the reproducibility of quantitative evaluation. Furthermore, as ECV fraction calculated by IC can be measured by equilibrium phase images alone, it is thus robust against artifacts and free from misregistration. Our results may contribute to improving the diagnostic ability of TETs, especially in diagnosing HRTETs and thymic carcinoma. We believe that ECV fraction measurement derived from contrast-enhanced DECT in the equilibrium phase will become commonly used for evaluating TETs.

Several limitations must be considered for the present study. First, the retrospective study design is a limitation of our study. Second, we included only a small number of TET patients from a single institute, which limited the statistical power and universality of the study. In addition, this study included no patients with type A thymoma, which has been reported to show high ECV fraction [23]. These limitations may have affected our results. A well-designed prospective study with a large number of cases and a wider range of tumor types is needed to confirm our findings. Third, IC derived from DECT may vary depending on the CT scanner, imaging quality which depends on patient body thickness, and contrast material injection protocol. Our results were obtained from a single-CT vender with a dual-layer DECT. Therefore, further evaluations should be conducted with other types of DECT scanners. Furthermore, it should be noted that differences in imaging parameters and the amount of administered contrast materials affect the results. Forth, the calculation of IC is based on subtle change between low kvp data and high kvp data, which may result in a large error. However, the error of the measured IC value has been reported to be limited [26], and we believe that the IC values in our results are reproducible. Fifth, IC was measured within the ROI placed on a single CT slice to evaluate the tumor and the results in this study could be affected by the heterogeneity within the lesion. Recent studies [27, 28] reported the usefulness of texture analysis for diagnosing TETs by evaluating volumetric and texture features such as irregular margin and heterogeneity associated with intralesional necrosis. Further investigations with volume-of-interest (VOI) analysis and radiomics approach are needed.

In conclusion, IC and ECV fraction derived from DECT are helpful in diagnosing TETs. High IC during the equilibrium phase and high ECV fraction are suggestive of thymic carcinoma.

## Abbreviations

TET: Thymic epithelial tumor
WHO: World Health Organization
FNA: Fine needle aspiration
IC: Iodine concentration
DECT: Dual-energy CT
ECV: Extracellular volume
LRTET: Low-risk thymic epithelial tumor
HRTET: High-risk thymic epithelial tumor
ICC: Intraclass correlation coefficient
ROC: Receiver-operating characteristic
SD: Standard deviation
AUC: Areas under the ROC curve
CI: Confidence interval
